# “This changes things”: Children, targeting, and the making of precision

**DOI:** 10.1177/00108367211050274

**Published:** 2021-10-22

**Authors:** J Marshall Beier

**Keywords:** childhood, drones, legitimacy, noncombatants, precision, subjecthood

## Abstract

Avoidance of civilian casualties increasingly affects the political calculus of legitimacy in armed conflict. “Collateral damage” is a problem that can be managed through the material production of precision, but it is also the case that precision is a problem managed through the cultural production of collateral damage. Bearing decisively on popular perceptions of ethical conduct in recourse to political violence, childhood is an important site of meaning-making in this process. In pop culture, news dispatches, and social media, children, as quintessential innocents, figure prominently where the dire human consequences of imprecision are depicted. Children thus affect the practical “precision” of even the most advanced weapons, perhaps precluding a strike for their presence, potentially coloring it with their corpses. But who count as children, how, when, where, and why are not at all settled questions. Drawing insights from what the 2015 film, *Eye in the Sky*, reveals about a key social technology of governance we have already internalized, I explore how childhood is itself a terrain of engagement in the (un)making of precision.

## Introduction

*Eye in the Sky*, a 2015 film from director Gavin Hood in which a drone strike on suspected members of the al-Shabaab militant group is hampered by the presence of a young girl within the projected kill zone, offers an instructive (if incidental) glimpse into the constitutive social terrains on which seemingly objective properties of some of the world’s most advanced weapons systems are at least partially founded. Fundamentally an exploration of the dilemmas posed in weighing utilitarian and deontological ethics, the film has been analogized to the famous “trolley problem” (see, for example, Cole, 2016; Crockett, 2016; Robson, 2020), presenting a scenario in which the life of the child comes to be measured against the lives of a far greater number of civilians expected to be killed if the strike is not carried out immediately. Tension builds as a 9-year-old Alia Mo’Allim (portrayed by Aisha Takow) unwittingly moves in and out of harm’s way while the intended targets are seen fitting two would-be suicide bombers with explosive vests and readying them to carry out a presumably immanent attack. Desperate to strike their targets from a watchful MQ-9 Reaper drone loitering in the sky above, British military officers struggle to attain an assessment of the risk to Alia low enough to satisfy political masters whose authorization is needed in order to proceed. Ultimately, the extent to which the child is within or without the weapon’s assessed lethal radius is what makes the strike alternatingly viable and unviable.

As a contemporary take on a familiar thought experiment in applied ethics, *Eye in the Sky* makes for compelling viewing. It reflects too on deeper ethical questions around war waged from afar in its depiction of a real-time web of actions and deliberations among powerful figures in the United Kingdom, the United States, Singapore, and China—all safe, secure, and far from the site of the violence they will each participate in wielding—before the unseen drone, remotely piloted from an air-conditioned trailer on the outskirts of Las Vegas, Nevada, dispatches its Hellfire missiles with devastating effect. The child, Alia, and the Reaper itself are key to these explorations, each giving important meaning to the scenario without which its deeper reflections would not be possible. Left unexplored, however, is an aspect of the relationship between child and weapon that is revealing of important indeterminacies in the seemingly objective properties of each. Key in this regard is how the child, once subjectively constituted as such, becomes determinant of what is normally treated as though an objective property of the weapon: the degree of its precision. In what follows, I draw on insights from the sociology of childhood in arguing that the very ascription of “child” is a terrain of engagement whose outcome bears directly on the constitution of precision as a social “fact” requisite to the perceived legitimacy of recourse to political violence. And though this is not something addressed directly by *Eye in the Sky*, the film is nevertheless revealing of it—perhaps less in the filmmakers’ intent than in the social competencies we bring to viewing it—inasmuch as its narrative strategy relies on it.

## When things change

Set in 2015, *Eye in the Sky* opens with scenes of everyday family life in the mostly Somali Eastleigh suburb of Nairobi. A mother bakes bread in an outdoor oven on the grounds of a modest home while her husband makes a toy hoop for their 9-year-old daughter, Alia. The camera ascends to reveal danger beyond the walls that separate their small dusty yard from the street outside, where armed militias patrol. These images are soon placed in wider context by way of references to the 2015 massacre at Garissa University College and the deadly 2013 Westgate Shopping Mall attack, video from which is shown in connection with fictionalized news coverage of the al-Shabaab killing of a man who we learn had, per the storyline, been working with British and Kenyan intelligence services to disrupt recruitment of radicalized Westerners. The scenes that follow introduce a cast of characters who, though spread around the globe, are connected in real time as they perform integrated roles in an operation to apprehend suspected members of al-Shabaab at a safehouse that happens to be adjacent to the home of Alia and her parents. From the outskirts of London, British Army Colonel Katherine Powell (Helen Mirren) commands the operation while in direct communication with field agents in Kenya as well as the Kenyan military. In Las Vegas, Second Lieutenant Steve Watts (Aaron Paul), a US Air Force drone operator who will soon find himself remote piloting the Reaper loitering unseen high above Eastleigh, explains to a colleague that he joined the Air Force because he had accumulated college debt. Real-time intelligence analysis is provided by still others in the loop from Pearl Harbor, Hawaii. In an early scene, British Lieutenant General and Deputy Chief of Defence Staff, Frank Benson (Alan Rickman), shops for a doll for his granddaughter before the operation gets underway; in the next scene, we see Alia in her room with a doll of her own sitting beside her on her bed. A few kilometers away, a young al-Shabaab recruit arriving in Nairobi from the United States comes under immediate surveillance as he is met at the airport by another man.

A briefing from Colonel Powell through video link to Creech Air Force Base in Nevada connects these and other pieces of the drama about to unfold. We learn that in addition to the new recruit from the United States, Powell has intelligence that another has arrived from the United Kingdom and both will be received at the safehouse in Eastleigh by two key members of al-Shabaab who are targets of the operation. A third target is the wife of one of the wanted al-Shabaab men, a woman whom Powell describes as a British national with a “troubled childhood, converted at 15 . . . radicalized in a West London mosque” [0:09:10]. Lieutenant Watts’ Reaper is to be the “eye in the sky” for Kenyan special forces who will be tasked with surrounding the safehouse and carrying out the capture of the occupants. The mission takes an abrupt turn, however, when it comes to light that the two new recruits are being fitted with bombs and readied to carry out a suicide attack. Alan Rickman’s General Benson sums up the moment of this revelation with a punctuating line, featured also in the film’s trailer: “Well, this changes things” [0:34:15]. As Powell’s threat assessor estimates the impending suicide attack could kill some 80 people, the operation does indeed change: from a capture mission to a targeted killing of everyone in the al-Shabaab house using the Reaper’s precision-guided Hellfire missiles. But for senior members of the UK government who have joined Benson to watch events unfold, political and legal considerations become paramount. As Powell seeks authority to order the strike, concern is expressed about the implications of carrying out a deadly missile attack on two British subjects and an American citizen in a friendly country—the legal issues are challenging but the political considerations, it seems, even more so. Still, these complications are eventually (if reluctantly) accepted and the strike is authorized to proceed. All appears settled when Alia suddenly comes into view of the Reaper’s powerful camera, setting up at a table inside the Hellfire’s blast radius to sell her mother’s bread. With apologies to the fictional General Benson, this is the development that *really* changes things.

All of this sets the stage for the specific dilemma explored in *Eye in the Sky*, but much more is at work in the worlds beyond the film on which its intelligibility depends. Perhaps most fundamentally, it belongs to an historical moment in which material and cultural technologies of precision warfare are deeply embedded in popular consciousness and, as such, condition understandings and expectations of relatively costless war (Beier, 2003; Zehfuss, 2010). Since the technocultural watershed of the 1991 Gulf War, which brought discourses and semiotics of “smart bombs” and “surgical strikes” to popular consciousness, this has been reflected along two distinct but related lines. First, the advent of reliable standoff weapons, capable of precisely hitting targets from great distances, has seen publics of the technologically advantaged Global North, in particular, come to expect low (in historical terms) levels of risk to their own forces. But it is in three decades of carefully cultivated claims of meaningful separation of combatants and noncombatants that we have witnessed the most profound implications of weapons billed as so precise that they have sustained the idea of a new standard of legitimate recourse to violence, one premised on minimal incidental harm to civilians. This is the broader cultural context in which the idea of armed drones has moved from the spectacular to the everyday in global public imaginaries over the decade and a half since the first widely publicized Hellfire-armed MQ-1 Predator—forerunner of the Reaper—strikes of the mid- to late 2000s. William Walters (2014) places these developments in the same cultural context, with broad acceptance of the idea that drones are precision strike capable that has, like the popular reception given Tomahawk cruise missiles and laser-guided bombs of the 1990s, had the corollary effect of producing an expectation of meaningful discrimination, setting a high standard for legitimacy that does not easily abide the incidental killing of recognized noncombatants.

These are the very sensibilities that animate, in one way or another, the actions of the various characters in *Eye in the Sky* and they are among the requisite cultural competencies audiences bring to the film as well. In popular cinema, as in the world from which it draws inspiration, the perceived capability to strike targets with surgical precision exacts a toll on legitimacy, with use of force more likely to be judged excessive where it results in civilian injury or death (Schmitt, 2005: 457). The 1991 war on Iraq, for example, saw a 10-day pause imposed on bombing of Baghdad following an instance of significant collateral damage (Reeve, 2013: 57), even as precision-guided munitions were being heralded as enabling a more legitimate practice of warfare. Then and in the years since, a few privileged states have benefited from juxtaposing claims to this capability with the inherently indiscriminate practices of political violence witnessed from and/or imputed to global adversaries. In this sense, the *rhetorical* technologies of precision have become at least as consequential as those delimiting performance characteristics of advanced weapons themselves. Those who wield both must consequently be attentive—as, indeed, they very much are—to the potential that any failure to manage perception in this way could preclude the weapons’ use altogether. With particular attention to how it has been propagated in media and popular culture, Philip Sabin (2018) describes this as the “new paradigm” of precision, highlighting how “rising expectations regarding the avoidance of collateral damage” may raise the political threshold for popular tolerance of the use of advanced airpower (p. 25).

A perverse consequence of these developments is that viable recourse to political violence itself emerges as an important object of protection under this new paradigm. Writing in the professional journal of the US Air Force, Merrick Krause (2015) cautions that “sensitivity to collateral damage will hinder the use of airpower and may put leaders in a position where dramatically handcuffed airpower is insufficient to be decisive” (p. 50). Similarly, in a report published by the Royal Australian Air Force’s Air Power Development Centre, Sanu Kainikara (2009) suggests that collateral damage is a “vulnerability” bedeviling the attacker inasmuch as it “could lead to political constraints being placed on the employment of air power that in turn will diminish its effectiveness” (p. 59). Military professionals are thus keenly aware of the *political* dimension of precision and of the potential for advanced weapons technologies to mitigate the mission-limiting effects of popular sensitivity to collateral damage, so long as they perform as intended (see, for example, Lambert, 2018; Maybaumwisniewski et al., 2004). Among the mechanisms employed to manage this, the US Air Force has for close to two decades relied on a software tool, the Fast Assessment Strike Tool—Collateral Damage (FAST-CD), that weighs parameters of terrain, attack angle, weapon characteristics, and more to generate projections on the anticipated damage area of a proposed strike (Graham, 2003), thereby enabling estimates of probable harm to noncombatants that can aid in deciding whether it is advisable to proceed with an attack. We can gather from claims of lower risk of collateral damage that appear in promotional materials and even patent filings for new weapons systems that arms manufacturers also recognize the need to be responsive.

The arms technologies portrayed in *Eye in the Sky* operate in this rhetorical space of managed perception. For the handful of advanced militaries that possess them, high-end weaponized drones like the Reaper are valued for their stealth, properties of endurance that permit long loiter times over target areas, and sophisticated surveillance capabilities. Particularly relevant in the context of *Eye in the Sky*, they have been touted as a solution to the problem of collateral damage when engaging targets in populated areas (Clouet, 2012: 182). The material precision of their strikes, however, comes down to the weapons payloads they carry. While the Predator is limited by its ability to embark the Hellfire only, the Reaper is capable of carrying a mixed payload of Hellfires and even more devastating GBU-12 Paveway laser-guided bombs. At 500 pounds, the GBU-12 produces a larger casualty area than the 100-pound Hellfire. The latter, then, is the more “surgical” of the two. Yet, even with its smaller explosive yield, the Hellfire has a “lethal radius” of 15 meters and a “wound radius” of 20 meters (Chamayou, 2015: 141–142). These, of course, are *ceteris paribus* characteristics that do not in themselves account for variables like terrain or the placement of structures that might variously blunt, amplify, or direct the weapon’s blast. “Bugsplat,” the dark sobriquet for the irregularly shaped anticipated damage maps generated by FAST-CD, reflects this complexity as well as the importance of distinguishing between accuracy (as a weapon “arriving” at an intended point in space) and precision (as the excision of an intended target). Beyond this, the algorithm behind FAST-CD encodes its own assumptions, vagaries, and a necessary politics inasmuch as it is, after all, a tool intended to assist commanders in satisfying ethical requirements as a means to facilitate a strike (Emery, 2020).

This begins to reveal something of the indeterminacies in precision, at least in its popular framings as a technical problem of accuracy. Measured on the basis of a weapon’s circular error probable (CEP), accuracy—that is, reliably centering an intended target—is an important but by no means the sole element of precision. What is more, even the Hellfire’s assessed accuracy of a few meters is more fraught than that aspect alone might suggest since the standard for determining CEP is not that the weapon always arrives within that range but that it does so 50% of the time (Sheedy, 1988). Taken together with the extent of and exigent variations in lethal radius, this paints a very different picture than the one conjured in talk of “surgical strikes” and claims to legitimacy premised on meaningful discrimination between combatants and noncombatants. Put simply, there is much more contingency in the material production of drone strike precision than is betrayed in attendant rhetorics or than may be sustained in public imaginaries.

In prepared remarks delivered at the Wilson Center in 2012, then-Obama Administration Deputy National Security Advisor for Homeland Security and Counterterrorism, John Brennan (2012), made out the now-familiar case for targeted drone strikes as enabling a more ethical practice of warfare, arguing that

compared against other options, a pilot operating this aircraft remotely—with the benefit of technology and with the safety of distance—might actually have a clearer picture of the target and its surroundings, including the presence of innocent civilians. It’s this surgical precision—the ability, with laser-like focus, to eliminate the cancerous tumor called an al-Qa’ida terrorist while limiting damage to the tissue around it—that makes this counterterrorism tool so essential.

Made explicit by Brennan, the operant metaphor of the surgical strike turns on the precise excision of a targeted malignancy without causing harm to surrounding tissues (Beier, 2006: 271). In actual surgical practice, this depends on the skill of the surgeon to cut precisely and, indeed, that may be how the metaphor is most apt to be decoded. But surgical precision depends too on the properties of the tools at hand—even the most talented surgeon will lack for precision without the right instruments.

Analogously, the Hellfire missiles carried by the fictional Lieutenant Watts’ Reaper are just too blunt an instrument to kill the intended targets while preserving Alia from harm. But where the analogy breaks down is when we begin to look more closely at the social world of the scenario presented to us by *Eye in the Sky* and, equally important, our own social worlds and the part they play in our making sense of the dilemma sketched in the film. For the surgeon, flesh is flesh, whether healthy or malignant. Likewise, the skill of the surgeon and the material properties of the instrument are what they are. In the case of the surgical strike, however, the skill of the drone operator and the properties of the weapon may be definable (within ranges of variation that are more or less quantifiable) but here the properties of the flesh are decidedly indeterminate. They are of the social worlds of the film and of the viewer. The weapon is precise enough when Alia walks beyond the kill zone, but not so when she (re)enters its lethal radius. Although the material properties of its CEP remain constant, it is, to return to the analogy, as if the healthy flesh were suddenly to become so hopelessly enmeshed with the target of the surgeon’s knife as to thwart the operation.

## Changing weapon-things

Attendant rhetorical technologies notwithstanding, the likelihood of collateral damage is a wholly anticipated dimension of precision strikes (Schmitt, 2005: 453), and one which calls for deft management of political consequences. This is evident in the way some military professionals seem to position recourse to political violence as itself an object of protection—one might even say a referent object of security—in their expressions of concern about how it is affected by the “new paradigm” of intolerance of civilian casualties. The complicated array of considerations weighed by FAST-CD is but one indication of the tall-order task that confronts them in this regard. But no matter how sophisticated the software, there remains much to exceed it and to confound even the best counsel of military lawyers and others involved in the risk-assessment exercise. As Michael N. Schmitt (2005) notes,

Various factors contribute to collateral damage and incidental injury—incomplete or incorrect knowledge about the target, failure to anticipate how civilians will be affected, inaccuracy, an inability to precisely measure the force applied to ensure no more than necessary is used, and restriking a target because it cannot be reliably determined whether it has been sufficiently neutralized. Precision improves the “quality” of attack in each of these regards. (p. 457)

Many other material factors also influence lethality (see Geneva International Centre for Humanitarian Demining (GICHD), 2017), underscoring that FAST-CD is an interpretive exercise in the weighing of probabilities. We might add to these various considerations the manner in which the noncombatant is constituted as such. This, after all, is also a key determinant of collateral damage. What is more, I would like to suggest, it is a key determinant of precision as a social fact also.

Precision has different faces, one of which resides in the world of material properties and capabilities and one arising from the intersubjective realm of popular expectation and perception. In the former sense, it is a technical problem of achieving the meaningful separation of combatants from noncombatants. Precision in the latter sense of expectation and perception reverses this. Here, it is not a matter of adherence to the norm of discrimination being produced by the precision of the strike but, rather, the precision of the strike *as a social fact* is produced by perceived adherence to the norm of discrimination. The actual material properties of the weapon, apart from all that is contingent about them, do not change, of course. So too, the many variables of context with which the FAST-CD assessors must grapple are, even for all their own indeterminacy, functions of the material world. But the popular decoding of the strike—and, by extension, of the weapon—as variously precise or imprecise is continuously negotiated intersubjectively in its articulation with other social facts. This lack of fixity reveals a dimension of precision that is always political, interpreted, and contestable, even as it is indispensable to the (perceived) legitimate wielding of force. This is one consequence of the political trading on claims of precision and of meaningful discrimination that has figured so prominently in popular perceptions of the (il)legitimacy of organized political violence for the last three decades.

“‘Precision’ in terms of effectively hitting the target,” as Maja Zehfuss (2018) astutely observes, “is not the same as ‘precision’ in terms of not hitting anything else” (p. 67). But who or what is the anything else? Most fundamentally, it is both civilians and critical civilian infrastructure which, when brought to harm in an attack, constitute collateral damage. However, in the rhetorical technologies by which precision is constituted as meaningful discrimination between combatants and noncombatants and, in turn, underwrites claims of legitimacy or illegitimacy of violence, it is harm visited on civilian bodies that is most liable to trade as political currency for some and political liability for others (see, for example, Pearlman, 2020). It is not the case, though, that any failure to discriminate will be read in the same way or with the same implications. And while the international legal principle of proportionality holds open the possibility for the killing or injury of noncombatants to be judged reasonable (provided it is unavoidable and reconcilable with a legitimate military objective), assessing what is proportional is an inherently subjective exercise in itself and one that turns as much on whose political projects are privileged as on who, precisely, the noncombatants might turn out to be. In the politico-cultural space in which the rhetorical technology of precision operates, dire human consequences come at greater political cost when visited on some bodies than on others.

This is the context in which the child, Alia, serves as fulcrum of the dilemma explored in *Eye in the Sky*. While the civilian decision-makers to whom she is beholden are consumed with foreseeable political consequences of killing an innocent child, Colonel Powell is preoccupied with resolving precision as a technical problem. To this end, she repeatedly pressures a risk-assessment officer (Babou Ceesay) to rework various assumptions and parameters input to FAST-CD, finessing the bugsplat map enough to back off from initial estimates and bring the “assessed” risk to Alia within acceptable parameters to get political authorization for the strike. In so doing, she also follows the reversed course of the social fact in respect of which the meaningful separation of combatants from noncombatants is determinative of the precision of the weapon, not the other way around. The problem is that the weapon simply is not materially precise enough in the sense that its projected blast profile (including but not limited to simple radius) exceeds what is necessary to kill the intended targets surgically—that is, without harming Alia. In pushing for manipulation of the assessed risk to Alia, Powell works to manage the social fact of the weapon’s precision to the point where the strike can be made politically viable. That she does so a priori is particularly significant inasmuch as we are able to glimpse how the achievement of precision *as a capability* may rely as much on the purposeful production of the social fact as on advances in the perfection of material arms technologies. And we see too how childhood, as a technology of governance, bears not only on how those seeking recourse to political violence may be limited in their ability to wield it and on how political meaning will be made of the outcomes of their actions, but how it actually affects what might otherwise be imagined as objective properties of a material “thing”—that is, of the weapon itself. More than the specific figure of Alia, childhood itself affects the practical precision characteristics of the weapon-thing.

## Childhood changes things

Perhaps more readily understood as being subject to governance, the idea of childhood as itself a technology of governance calls for some brief elaboration. Whether formalized in law and institutional arrangements or manifest in norms and conventions of everyday life, the lives and lifeworlds of children are governed through such things as graduated rights regimes, regulation of bodily autonomy, delegated responsibility for security and wellbeing, deferment of participation, and limitations on presence in social spaces. In these and other assemblages and practices, the child of hegemonic imagining is a presocial being defined by incapacity and deficit relative to the idealized adult subject—less a “human being” than “human becoming” (Uprichard, 2008). Although it has come under sustained critique from post-developmentalist psychology (see Burman, 1994) as well as the new sociology of childhood that emerged in the 1980s, children’s geographies, and the broader interdisciplinary Childhood Studies specialty (Aitken, 2001; James and Prout, 1990; Jenks, 1996; Qvortrup, 1994), this developmentalist-inspired understanding of childhood continues to comport well with popular attitudes toward children. It is in this sense that the dominant understanding of childhood may be understood as a “technology” in the governance of children inasmuch as it is key to the (re)production and policing of the boundary between childhood and adulthood and the unequal social relations of power it sustains. At the same time, children’s disenfranchisement from political life entails their political innocence and, therefore, a presumptive expectation of protection from harm, to which they are entitled as putatively presocial beings (Brocklehurst, 2006). This parallels but runs deeper than the combatant or noncombatant distinction, which similarly premises the claim to protection on the presumption of non-participation (Kinsella, 2006).

As a technology of *global* governance, childhood is regularly summoned as a powerful rhetorical resource in service of sovereign power, purposefully deployed in ways that variously undercut or enable political projects of the adult world. The ascriptions child, child-like, or childish, for example, affect not only who may advance a persuasive claim to protection but also whose political subjecthood is abided and whose is denied. Childhood is thus, in one sense, an epithet, a derogation of legitimacy that may be wielded against any claim to a political subject position, even by or against sovereign power (see, for example, Basham, 2015; Mills and Lefrançois, 2018). Embodied and in abject circumstances, however, the same dominant understanding of childhood affects the production of (il)legitimacy in ways felt much more viscerally. As quintessential innocents and civilian noncombatants *par excellence*, children function as the evocative “emotional scenery” (Brocklehurst, 2015: 32) of conflict zones. Images of child victims of violence do the work of delegitimizing the acting subjects of that violence and the political projects they may seek to advance (Beier, 2018; Berents, 2019). As Katrina Lee-Koo (2018) argues, “the power of these images derives from their capacity to prompt simple narratives of global politics built upon binary constructions of ‘us’ and ‘them,’ ‘right’ and ‘wrong’ and ‘good’ and ‘bad’” (p. 49). This, in turn, relies on dominant ideas about childhood as the arbiter of where various political subjects are located in these binaries, as determined by their implication in violence visited on child bodies—violence that cannot easily be abided as legitimate but which may underwrite the claimed legitimacy of retribution. As Lee-Koo (2020) puts it, “The value of children’s suffering is in scripting state identity and emboldening state action” (p. 29).

Childhood is similarly at work in how legitimacy is governed in specific acts of political violence and, by extension, in the production of the social fact of precision. This demands that we think beyond the material properties of this or that weapon’s assessed accuracy. As Lucy Suchman (2020) argues, “as long as the ‘accuracy’ of the weapon is measured by the relation of lethal force to its designated object, the most vital question is left outside of the frame” (p. 183). For Suchman, “the fundamental question is how a target comes to be designated as such in the first instance, and within what regimes of historic injury and future accountability.” And it is likewise important to understand how those excluded as candidate targets come to be so assessed. Christiane Wilke (2017) shows how civilians are not merely “found” but, rather, are read as such (or not) through the mediating influences of the assumptions military personnel make about contexts, threat, and the ways in which different bodies are coded. Childhood is a coding that does important meaning-making work in this regard, but it too is constituted in the intersectionality (Crenshaw, 1989) of identity codings that include, among other things, race and gender. Colonial relations of power, shot through with raced, gendered, and other ascriptions, locate some children outside of the norms of childhood and consequently beyond presumptive political innocence and the relative protections it might confer (Liebel, 2020).

In what she terms “unchilding,” Nadera Shalhoub-Kevorkian (2019) shows how this works to place some children squarely within the ambit of sovereign power’s “gunsights,” at times quite literally. We see something of this in the ascription “military-age male,” synonymous with “combatant” and at times applied even to pre-adolescent boys (Shoker, 2021), while a young girl is much more likely to be read unambiguously as “noncombatant.” Produced in the reading of an encounter in real time, this is arbitrary not only in the sense of the underlying assumption that all males of a certain age are combatants but also in the estimation of who, in a given circumstance, seems to fit those criteria. Hugh Gusterson (2016: 67) points out how drone operators may assess something as simple as a gathering of individuals or what they interpret as those individuals’ participation in prayer to be confirmation that they are also insurgents and, thus, legitimate targets. The civilian, like the combatant, is produced as social fact through an interpretive process that relies heavily on other social facts that lend variously to political countenance or repudiation of the violence visited on specific bodies. As Krause (2015) observes, “[t]he will of both the public and elected leadership is influenced by the number and type of casualties, depending upon a number of factors, including whether or not the casualties are civilian, children or adults, women or men . . .” (p. 48). Of these, Wilke (2017) describes childhood as a “credible proxy and marker of likely civilian status” (p. 1053).

*Eye in the Sky* relies on our reading of childhood in just this way. Alia, a young child and a girl, reliably presents as a civilian noncombatant, innocent and deserving of protection from harm. As the film’s main political characters deliberate on whether it is justifiable to order the drone strike with Alia in harm’s way—assessed, we learn, to be a 65%–75% [1:00:25] probability of fatal injury and a certainty of severe injury—General Benson, clearly frustrated at what he perceives as their dithering, gets straight to the crux of the matter: “I hope the fact that she’s a sweet little girl isn’t clouding your judgement” [1:01:19]. As the debate proceeds, it turns out that childhood does indeed loom large in the political calculus that is unfolding. Benson’s framing of “a sweet little girl” calls attention to Alia’s political innocence, gleaned from the intersectionality of age and gender, and it seems he is correct in assessing its moral weight even if he is perhaps less moved by the political significance of that. Other characters soon make the point abundantly clear. Confronted on the projected cost of 80 lives if Alia is to be spared harm, Parliamentary Under-Secretary of State for Africa Angela Northman (Monica Dolan) responds, “Frankly, politically, I’d rather point to al-Shabaab as murderers of 80 people shopping than have to defend a drone attack by our forces that kills an innocent child” [1:04:19]. It is an assessment shared by Attorney General George Matherson (Richard McCabe): “If al-Shabaab kill 80 people, we win the propaganda war. If we kill one child, they do” [1:04:41].

These scenes and the central storyline of *Eye in the Sky* work only because of what we already “know” about childhood as a technology of governance. We bring the same competencies to decoding the movie as are popularly drawn upon in assessing the social meaning of “real-world” events. When, in 2017, then-US President Donald Trump premised strikes against Syria on what he described as the killing of small children and “beautiful babies” (see Harding, 2017) in a gas attack attributed to the Asad regime, that too counted on attentive publics decoding ideas like “civilian,” “child,” and even “surgical strike” in reliable ways that work to similar effect across myriad contexts and settings. With high numbers of civilian casualties in the 2014 Gaza War, for instance, Israel drew special condemnation for the killing of four children playing on a beach and, revealingly, seemed at pains to exonerate itself of wrongdoing in this particular instance (see Beaumont, 2015). Through just over 2 weeks of air strikes in May 2021, the killing of dozens of Palestinian children in what was characterized as an “AI war” of “precision strikes” (Ahronheim, 2021) quickly became a focal point for international pressure—also felt by Israel’s allies (see, for example, Bacon, 2021)—to halt attacks on Gaza. A *New York Times* article, which noted a widely held sense that Israel’s use of force in the May 2021 crisis was disproportional, featured photos and brief personal stories of more than 60 children killed in Israeli air strikes, the youngest only 6 months of age (El-Naggar et al., 2021). Many were killed together with adults, but the political toll on legitimacy came to reside most poignantly in the images and accounts of child victims.

Similarly instructive is a CNN (2018) video report on a 2018 Saudi airstrike that killed 40 Yemeni children on a school bus. The reporting on this incident, which drew extensive worldwide media coverage, included shocking images and graphic eyewitness descriptions of the attack and its gruesome aftermath. It also noted that the bomb that hit the school bus (which was in the middle of a busy market in Saada, Yemen at the time) on 9 August 2018 was a MK82 GBU-12 “Paveway” laser-guided precision weapon—like those dispatched from Reaper drones—produced by US arms maker Lockheed Martin. The CNN report noted the weapon’s targeting accuracy is a particular point of pride for Lockheed Martin and a Lockheed Martin promotional video, replete with familiar semiotics of technologically advanced precision warfare, was featured in the CNN report. Importantly, the same CNN report noted that, in March 2016, a laser-guided MK84 bomb was used in a strike that killed 97 people in a market in Mastaba, Yemen and, in October 2016, a strike on a funeral hall in Sanaa, Yemen killed 155 people. In aggregate human terms, the strikes in Mastaba and Sanaa resulted in much higher death tolls than the attack on the school bus in Saada, but neither received the same extent of mainstream news coverage nor ignited weeks of sustained reflection on questions of responsibility for their dire outcomes, as did the bombing of the bus. Children have been killed together with adults in countless other strikes in Yemen and elsewhere, but a precision strike on something as iconically connected to childhood as a school bus generated a higher order of outrage despite having resulted in fewer civilian casualties than other such attacks. This tells us something important about what we might term the “practical” precision of the weapon at issue. It is likewise telling of what is reliably “known” about childhood and how this functions as a technology of governance in the making and unmaking of precision.

It is precisely these sorts of potential outcomes that confront the characters in *Eye in the Sky*, while under pressure to act (or not) before the moment of opportunity is lost. Just as the viability of the drone strike in the film waxes and wanes with Alia’s proximity to the projected blast area, real-world deliberations on whether to carry out an attack are likewise affected by the presence of children. The fictional Colonel Powell attempts to resolve the impasse that confronts her by insisting on manipulation of the bugsplat and demanding questionable interpretive leaps from her risk-assessment officer until they can be made to yield an estimate of harm to the child low enough to appease reluctant political masters, but she might just as readily have submitted “child” to interpretation had the filmmakers not framed Alia so unambiguously as such. Indeed, as Sarah Shoker (2021: 53–54) shows, drone operators themselves acknowledge that the constitution of “legitimate” targets routinely involves the subjective assessment of who is or is not a child, at times explicitly discussed in the instant of determining whether to dispatch a loitering drone’s Hellfire missiles (see Allinson, 2015: 121–123). That estimations of the danger posed to children might be finagled in various ways—in real-world moments of decision as much as in the dilemma portrayed in *Eye in the Sky*—only reaffirms the importance of childhood as a terrain of engagement affecting recourse to political violence. For audiences at the cinema as for global publics weighing the legitimacy of an actual drone strike, it is a key arbiter of precision and, by extension, of legitimacy.

## Conclusion: confounding precision

Childhood changes things. Specifically, childhood changes the practical properties of the weapon. The child in *Eye in the Sky* renders the weapon alternatingly precise and imprecise as she moves in and out of the target area. Objectively, the weapon’s material properties remain constant; it will destroy what it will destroy and kill whom it will kill within a defined radius and as affected by the sorts of variables weighed by FAST-CD, regardless of whether Alia is within or beyond its reach. And yet, in a very consequential way, the characteristics of the weapon, as precise or not in the sense of precision as a social fact, vacillate with the extent of the child’s proximity. We are used to thinking in terms of how advanced “hard” technologies and information-handling capabilities can render a weapon more precise in the narrowing of CEP and reduction of aggregate failure rates and even through better training, drilling, and policing of military professionalism. But as evocative of the innocent noncombatant par excellence, childhood is also a site of technological intercession affecting precision—in this case, a social technology applied in decoding the figure of the child. Precision as a property of the weapon is, in practical terms, contingent on the workings of childhood as a governance technology both in the indeterminacies around who may be recognized as a child and in response to the rather more random circumstance of children’s presence or absence within the CEP.

Just as significant, the weapon might be precise enough if someone other than Alia were to wander into the kill zone, depending upon who that someone may be. Who is a civilian or, as Thomas Gregory (2012) argues, whose lives are worthy of protection and whose may be marked for killing, is not at all as clear-cut as the combatant–noncombatant binary might be taken to imply. Importantly, though, childhood as a technology of governance is not an objective function of the material property of age. Rather, childhood, like precision, is a social fact instantiated in connection with other social facts. Equivocation, then, also takes place along the register of who is and is not recognized as a child. Alia’s gender alone is not enough, as we can see from the inclusion of an adult woman among the film scenario’s intended targets; her age alone is not enough, because a boy of her age in her part of the world might much more easily be marked as a combatant (Gregory, 2006; Shoker, 2021; Wilcox, 2017). Childhood matters, but it matters differently depending on how it intersects race, gender, and other constituent categories of subject identity formation which can position some children outside of childhood as hegemonically understood. It is Alia’s unambiguous coding as child that does this important meaning-making work and, no less, our own social competencies as viewers, drawing meaning from the totality of her intersectional identity, that have us read her character precisely this way.

Doing so reveals something of the work done by childhood itself—here, innocent childhood, outside of political subjecthood, and therefore, tragic death opening the possibility of the least legitimate sort of killing. Acknowledging that 40 people were killed means something different from saying 40 civilians were killed, and saying 40 children were killed is qualitatively different again. Each framing opens or forecloses political possibilities. A drone strike that kills 40 intended targets is intelligible as war; kill 40 civilians, and the preservation of legitimacy becomes contingent on the extent to which it can be framed as a tragic exigency of war; the killing of 40 children is likely indefensible in any framing. Childhood has a particular ontological and phenomenological significance that makes its invocation or contestation politically consequential in ways substantively different from subjects otherwise socially located. A great deal of political work is consequently devoted to defining who is and is not (or was and was not) a child in war zones and which acts of violence are, therefore, more egregious than others. This speaks also to how some bodies are made legitimate—or at least much more legitimate—targets.

But whatever the indeterminacies of its constitution, the identity ascription “child” confounds precision, rendering more or less consequential the material properties of the object that is the weapon. It is therefore useful to think in terms of both material and practical measures of precision. Material precision is affected by essentially fixed properties of accuracy and the challenging array of variables from which FAST-CD derives its bugsplat maps. It turns too on information gathering and processing, assessment, target selection, as well as relative qualities of competence, proficiency, and professionalism in wielding arms. Practical precision, however, is a distinction that recognizes the sociopolitical context in which the weapon is used and how the meaning of that use is made in connection with larger and more complex webs of social meaning less amenable to measurement. By way of analogy, a bullet is more precise traveling through air than through water. We may say too that it is more precise on the battlefield than in a crowded market, where the sociopolitical costs of it striking an unintended target are apt to be more acutely felt. The weapon itself is thus revealed as merely one part of a complex technological system made up as much by cultural technologies of governance as by arms technologies. Childhood is key among these governance technologies—an intervening social technology that mediates the precision of the weapon as a social fact.
